# Health risk assessment of lake water contaminated with microcystins for fruit crop irrigation and farm animal drinking

**DOI:** 10.1007/s11356-023-27914-1

**Published:** 2023-06-09

**Authors:** El Mahdi Redouane, Zakaria Tazart, Majida Lahrouni, Richard Mugani, Sara Elgadi, Hamza Zine, Soukaina El Amrani Zerrifi, Mohammed Haida, José Carlos Martins, Alexandre Campos, Khalid Oufdou, Vitor Vasconcelos, Brahim Oudra

**Affiliations:** 1 Water, Biodiversity and Climate Change Laboratory, Faculty of Sciences Semlalia, Cadi Ayyad University, 40000 Marrakech, Morocco; 2 CIIMAR, Interdisciplinary Centre of Marine and Environmental Research, Terminal de Cruzeiros do Porto de Leixões, 4450-208 Matosinhos, Portugal; 3 Laboratory of Microbial Biotechnologies, Agrosciences, and Environment (BioMAgE), Labeled Research Unit-CNRST N°4, Faculty of Sciences Semlalia, Cadi Ayyad University, 40000 Marrakech, Morocco; 4 Laboratory of Agro. Food Technology and Quality, Regional Center for Agronomic Research of Marrakech, National Institute of Agronomic Research (INRA), 40000 Marrakech, Morocco; 5 Geology and Sustainable Mining Institute (GSMI), Mohammad VI Polytechnic University, 43150 Ben Guerir, Morocco; 6 Higher Institute of Nurses Professions and Health Techniques of Guelmim, 81000 Guelmim, Morocco; 7 Department of Biology, Faculty of Sciences, University of Porto, 4169-007 Porto, Portugal

**Keywords:** Microcystins, Raw water, Farm animals, Fruit crops, Bioaccumulation, Health risk

## Abstract

The health risks linked to the consumption of microcystin-accumulating crops have been increasing worldwide in toxic cyanobloom-occurring regions. The bioaccumulation of microcystins (MCs) in agricultural produce at environmentally realistic concentrations is poorly investigated. In this field study, we assessed the health risks of MCs in raw water used for irrigating fruit crops (bioaccumulation) and watering farm animals in the Lalla Takerkoust agricultural region (Marrakesh, Morocco). Thus, MCs were extracted from water and fruit samples and quantified by enzyme-linked immunosorbent assay in order to calculate the health risk indicators. MCs posed a high health-risk level to poultry and horses, with estimated daily intakes (EDI) being 14- and 19-fold higher than the recommended limits (3.1 and 2.3 μg MC-LR L^−1^), respectively. Furthermore, pomegranate posed the same level of risk, with EDI being 22- and 53-fold higher than the limit dose (0.04 μg MC-LR kg^−1^) for adults and children, respectively. There was an urgent need for guidelines regarding water use and management in MC-polluted areas, besides the setup of nature-based tools for toxin removal from raw water used in farming practices. Moreover, MCs could contaminate the human food chain, which implies further investigations of their potential accumulation in livestock- and poultry-based food.

**Supplementary Information:**

The online version contains supplementary material available at 10.1007/s11356-023-27914-1.

## Introduction

The expansion of toxic cyanoblooms in surface waters raises concerns worldwide due to their ability to produce and release cyanobacterial toxins (cyanotoxins) (Merel et al. 2013). Microcystins (MCs) are cyclic heptapeptide cyanotoxins mostly produced by the bloom-forming cyanobacterium *Microcystis* and widely distributed across 108 countries around the world (Harke et al. 2016). They pose an ever-increasing biohazard to humans and livestock as potent hepatotoxins and tumor-inducing cyanopeptides (Xu et al. 2020; Hernandez et al. 2021; Shi et al. 2021). Furthermore, MCs are known to be phytotoxic and stress-inducing compounds, causing impairments in plant growth and losses in agricultural yield (Lahrouni et al. 2016; Campos et al. 2021).

Intracellular MCs are released in water bodies after cyanobacterial cell death and lysis (Ross et al. 2006). They persist in water for several months at concentrations ranging from less than 1 μg L^−1^ to 29000 μg L^−1^ (Zastepa et al. 2014; Massey et al. 2020, Pham et al. 2021). Thus, the World Health Organization (WHO) has set safety limits of around 1 μg L^−1^ and 12 μg L^−1^ of MC-LR in drinking water for long- and short-term exposures, respectively (WHO, 2020). Furthermore, MCs are transferred to the soil-plant system via contaminated irrigation water and can persist for several days (Machado et al. 2017; Redouane et al. 2019; Liu et al. 2021). The root system absorbs MCs, which are translocated and accumulated thereafter in edible organs of plants such as celery, pepper, and cabbage (Xiang et al. 2020). Their bioaccumulation poses health risks upon consumption of MC-accumulating crops (Redouane et al. 2019). Therefore, the WHO has recommended a daily limit of 0.04 μg of MCs per kilogram of body weight (WHO, 2020).

Crop contamination by MCs may occur via several pathways, including (a) the inflow of cyanobacteria-contaminated surface waters to ground waters (Mohamed et al. 2022); (b) the application of cyanobacterial biomass as biofertilizer in agricultural practices (Chen et al. 2006; Xiang et al. 2020); and (c) the occurrence of MC-producing cyanobacteria in groundwater and agricultural soils (Ye et al. 2021; Zhang et al. 2021). The use of MC-contaminated irrigation water may intertwine with the previous contamination pathways and raise the health risk due to the consumption of MC-contaminated crops. Nevertheless, there are no official reports about deaths caused by exposure to MCs to date, except for the Caruaru incident related to their direct contact via renal dialysis in 1996 in Brazil (Azevedo et al. 2002). Human health concerns are often linked to chronic exposure to lower doses of MCs targeting many vital organs (Massey et al. 2018). Exposure to MCs mainly occurs through the ingestion of contaminated food and water and the inhalation of the aerosolized toxin (Massey et al. 2018; Breidenbach et al. 2022). On the other side, animal deaths and severe intoxications linked to MCs have been reported in previous years (Mez et al. 1997; Wood et al. 2010; Dreher et al. 2019; Zhang et al. 2022).

Scarcity of water resources in arid and semi-arid regions is linked with global changes, and it is often coupled with the expansion of MC-producing cyanoblooms, as is the case in our current study, the semi-arid region of Lalla Takerkoust (Marrakesh, Morocco). In past decades, toxic bloom-forming *Microcystis aeruginosa* has been dominant in the eutrophic Lalla Takerkoust reservoir during the summer and autumn seasons (Oudra et al. 2001; Oudra et al. 2002; Douma, 2010; El Ghazali et al. 2010; El Khalloufi et al. 2013) (Fig. S3). Therefore, for the first time, we aimed to assess the sanitary risk linked to the ingestion of environmentally relevant concentrations of MCs in irrigation water (raw water), sourced from the Lalla Takerkoust lake-reservoir nearby, and also used for watering livestock and poultry. The real biohazard related to MC realistic concentrations in water bodies and agricultural produce is poorly tackled and investigated. Hence, the importance of this field study in filling the gap about their health risk under a realistic exposure scenario. Furthermore, we attempted to assess the human health risk of consuming MC-accumulating fruits in the agricultural perimeter of Lalla Takerkoust, which is constantly irrigated with MC-containing water.

## Materials and methods

### Study area and sample collection

Lalla Takerkoust town is located 35 km south-west of Marrakesh City in Al Haouz province, Morocco (31° 36′ N, 08° 02′ W, 619 m) (Fig. S1). It is a semi-arid pre-steppe area with an annual average temperature of nearly 20.37 °C (monthly-average temperature in January is 11.98 °C and that in July is 29.05 °C) and an annual average rainfall of 160.70 mm (NASA, 2021). The rainfall occurs mainly during the autumn and winter seasons, accounting for 71.25 % of the total annual precipitation (Fig. S2). Lalla Takerkoust reservoir was constructed by damming the N’fis river in 1935 for electricity production, potable water supply, and farmland irrigation (MEMEE 2008). The artificial lake is classified as eutrophic with nearly 70 Mm^3^ of volume and 1796 km^2^ of surface (Oudra et al. 2001), irrigating around 24000 hectares of fruit groves through canal irrigation (Rocade Canal) (MEMEE 2008). Rainfall scarcity is usually coupled with toxic bloom expansion in a semi-arid climate, and therefore, irrigation of fruit crops with MC-containing water is inevitable. Extracellular MCs were previously detected in the irrigation water sourced from the lake-reservoir of Lalla Takerkoust and reached a maximum concentration of 94.40 μg L^−1^ in December, 2005 (bloom period) (El Ghazali et al. 2011). More recently, in 2019, a *Microcystis* bloom collected from Lalla Takerkoust reservoir contained around 75.3% of *Microcystis aeruginosa* with 1622 μg mL^−1^ (fresh bloom) of total MCs (unpublished data). Agriculture is one of the most important pillars in the Haouz region, and related fruit products are traded far and wide. Moreover, livestock and poultry farmings are well-known in the Lalla Takerkoust region and consist mainly of cattle, sheep, horses, and chickens being watered with MC-containing raw water (water used for irrigation).

Eighty-nine fruit groves were selected for sample collection during the harvest season (mature fruits) in 2019 (Fig. S1, Table S1). The collected fruits were composed of pomegranate (*Punica granatum* L.) (27 groves), apricot (*Prunus armeniaca* L.) (20 groves), plum (*Prunus domestica* L.) (21 groves), grape (*Vitis vinifera* L.) (1 big farm), and olive (*Olea europaea* L.) (29 groves). Ten fruit samples (edible parts) for each species were collected randomly in each grove, mixed into one composite sample, and put into clean plastic bags. In addition, one-liter aliquots of irrigation water were collected twice a month (15-day intervals) during 2019 (from January to December). The water samples were collected from the main irrigation canal connected to the selected sampling groves and also used for farm animal watering. The water samples were preserved in sterile amber glass bottles and transported immediately to the laboratory for MC quantification. Fruit samples were washed with deionized water several times, cut into small slices, and stored at −20 °C (fresh form) for MC quantification. A second subset of apricots, plums, and grapes was freeze-dried for MC analysis since they are widely consumed in dry form.

### Microcystin extraction and analysis

Total MCs were determined in water and fruit samples using an enzyme-linked immunosorbent assay (ELISA) kit for congener-independent detection of total microcystins. The extraction and pre-purification of MCs from water and fruit samples were performed using amber vials and aluminum foil to avoid toxin loss by photolysis. Moreover, only glassware was used during the extraction procedure to avoid MC loss by adsorption onto plastic.

MC extraction and pre-purification from irrigation water were performed according to the procedure described in Triantis et al. (2016) and slightly modified. One-liter aliquots of water samples were filtered through glass-fiber membranes into clean glass bottles wrapped with aluminum foil. The pH within was adjusted to 5–8 when it was outside this range. Following this step, MCs were extracted from water with the use of reverse-phase extraction cartridges (LiChrolut® RP-18, 1000 mg/6 mL, Sigma-Aldrich, Munich, Germany). MCs were concentrated by passing water samples through the cartridges conditioned with 5 mL of methanol and 5 mL of deionized water. Following this step, the cartridges were rinsed with 10 mL of 20% aqueous methanol (*v/v*), and the MCs adsorbed were eluted with 3 mL of pure methanol. The eluates were vacuum-dried at 40 °C using a CentriVap vacuum concentrator (Labconco, Kansas City, MO, USA), and residues were redissolved in 1 mL of deionized water and stored at −80 °C until ELISA analysis. Extracellular MCs were only considered since no intact cyanobacterial cells (intracellular MCs) were observed in irrigation water from irrigation canals.

MC extraction from fruit samples was performed as described by Corbel et al. (2016), with slight modifications. Aliquots of 5 grams (in triplicate) of edible parts were ground in liquid nitrogen and then homogenized with 20 mL of 75% (*v/v*) aqueous methanol. Fruit slurries were sonicated at 40 kHz in an ice bath for 5 min using a sonicator probe (Hielscher, Teltow, Germany), and subsequently kept overnight at 4 °C for a maximal yield of MCs. Afterwards, all slurries were centrifuged (6000 ×g) at 4 °C for 10 min, and pellets were re-extracted twice as before. All supernatants were pooled, and the MCs within were pre-purified following the same procedure described in the “Microcystin extraction and analysis” section.

Total MCs were quantified by ELISA using the Eurofins Abraxis Microcystins-ADDA ELISA kit (Warminster, PA, USA) for quantitative and sensitive congener-independent detection of microcystins and nodularins, with a detection limit of 0.1 μg MCs equivalent L^−1^. The ELISA analysis was performed according to the procedure described in the user’s manual provided along with the kit. The absorbance was read at 450 nm using a RT-2100 Microplate Reader, Version 2.0e (OPTIC ivymen® SYSTEM, Guangdong, China). A certified standard of MC-LR was used for the calibration curve construction (0, 0.15, 0.4, 1, 2, and 5 μg L^−1^). A positive control of MC-LR was used (0.75 μg L^−1^), and the ELISA analysis was valid when obtaining a close value (0.75 ± 0.185 μg L^−1^). MCs in water and fruit samples were quantified in duplicates, their concentrations were determined using the standard curve, and they were expressed as micrograms of MCs per liter of water (μg L^−1^) or per kilogram of fresh weight (FW) or dry weight (DW) (μg kg^−1^ FW/DW).

### Health risk assessment

To estimate the potential human health risk associated with the consumption of MC-accumulating fruits, we used equation 1 to calculate the estimated daily intake (EDI) of MCs in fruits (μg MCs kg bw^−1^ d^−1^; bw: body weight) (Jia et al. 2018):

1 $$\mathrm{EDI}={~}^{\left({\mathrm{C}}_{\mathrm{fruit}}\times \mathrm{ADI}\right)}\!\left/ \!{~}_{\mathrm{AW}}\right.$$

C_fruit_ is the MC concentration in fruits (μg MCs kg^−1^); ADI is the average daily intake of the contaminated fruit (kg d^−1^); AW is the average weight (kg).

We assumed that an average-sized adult of 60 kg and a child of 25 kg consumed different amounts of fruits on a daily basis, as reported on the Food and Agriculture Organization of the United Nations website (FAO 2019) (Table S3). A risk quotient (RQ) was calculated using Equation (2) (Xiang et al. 2020) to determine the factor exceeding the tolerable daily intake (TDI) of MCs in contaminated fruits. The TDI was set at 0.04 μg kg^−1^ bw d^−1^ by the WHO (WHO, 2020).

2 $$\mathrm{RQ}=\mathrm{EDI}/\mathrm{TDI}$$

A risk level was evaluated based on RQ values; RQ < 0.1 indicates a low health risk, 0.1 ≤ RQ ≤ 1 represents a moderate health risk, and RQ > 1 indicates a high health risk (Xiang et al. 2020).

We have also assessed the sanitary risk associated with the consumption of MC-containing irrigation water used as drinking water for farm animals (livestock and poultry). According to the Australian and New Zealand Environmental and Conservation Council (ANZECC), MC-intoxication is likely when the TDI for livestock (cattle, sheep, and horses) and poultry (chickens) is exceeded (Table S2) (ANZECC 2000). The RQ for livestock and poultry was calculated using Equation (3):

3 $${~}^{\mathrm{RQ}={\mathrm{C}}_{\mathrm{water}}}\!\left/ \!{~}_{\mathrm{TDI}}\right.$$

C_water_ denotes the concentration of MCs in the consumed water (μg L^−1^), and TDI denotes the tolerable daily intake of MCs in water for each animal category (Table S2). A risk level was evaluated similarly for humans as described above.

### Statistical analysis and data processing

**Fig. 1:**
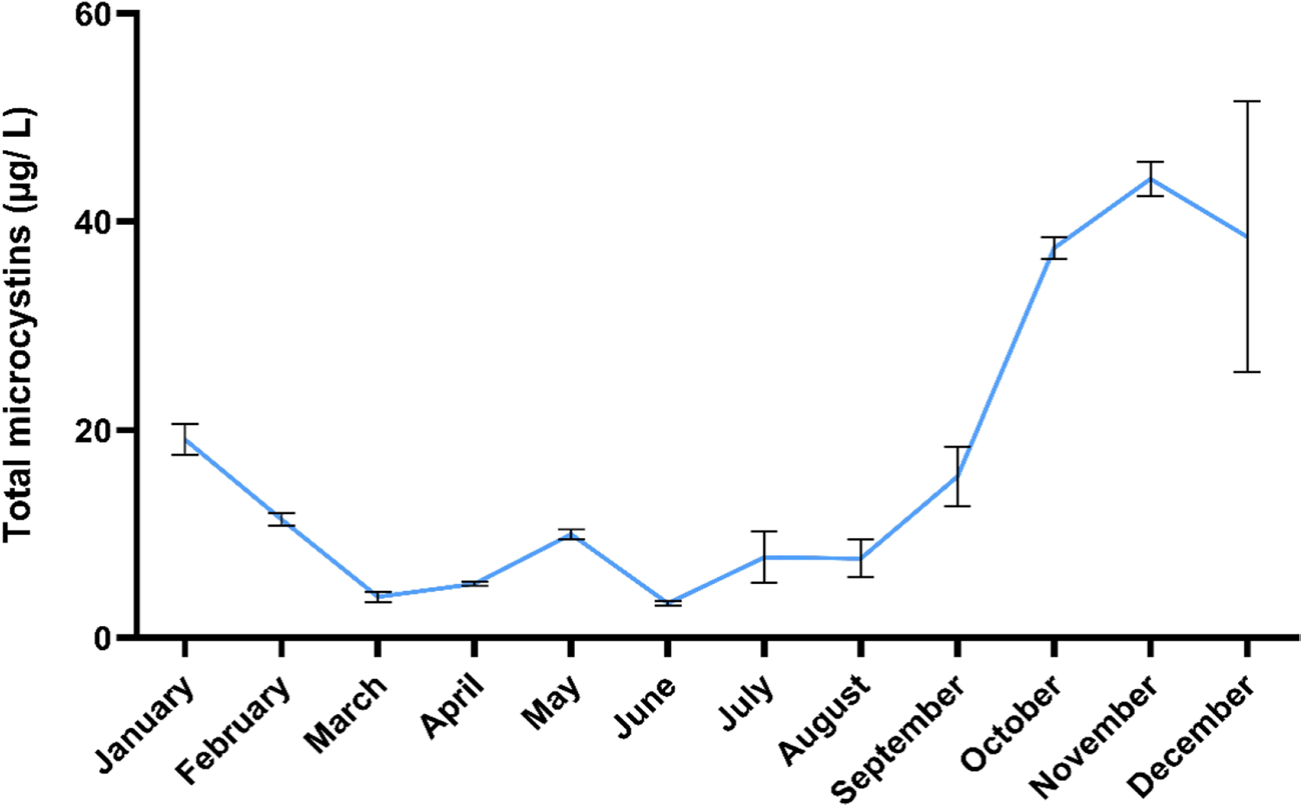
Monthly content of microcystins (μg L^−1^) in irrigation water sourced from the Lalla Takerkoust reservoir in 2019

**Fig. 2:**
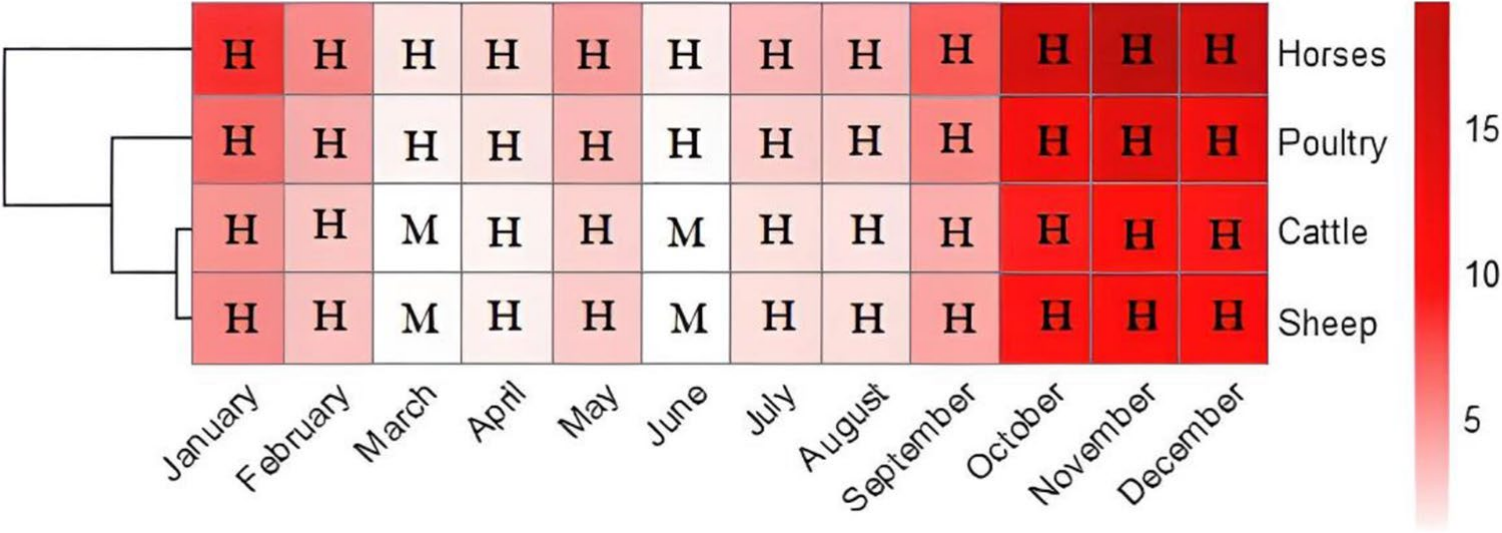
Heatmap of monthly-risk quotients and risk levels (H: high, M: moderate) related to microcystins in water used for watering livestock (horses, cattle, sheep) and poultry in 2019

The data were subjected to a one-way analysis of variance (ANOVA) using SPSS software, version 22.0 (SPSS Inc., 2013, Chicago, IL, USA), and the criterion for statistical significance was set at 5%. Figs S1 and 3 were drawn using QGIS software, version 3.16.9 (QGIS Development Team, 2020, Hanover, Germany). Line and grouped bar graphs (Figs. 1 and 4) were drawn and edited using GraphPad Prism 8 software, version 8.0.2 (263) (GraphPad Software, Inc., 2019, San Diego, CA, USA). Health risk heatmaps (Figs. 2 and 5) were drawn using R software version 3.6.2 (R Foundation for Statistical Computing, 2021, Vienna, Austria). The data was presented as the mean standard deviation (*n* = 3).

## Results and discussion

### Microcystin occurrence in irrigation water

The occurrence of cyanoblooms and their toxic metabolites is increasing in inland freshwaters worldwide used for drinking and irrigation purposes (Merel et al. 2013; Xiang et al. 2019). MCs were detected in irrigation water with monthly-average concentrations ranging from 3.33 to 44.12 μg L^−1^ in 2019 in the Lalla Takerkoust Lake (Fig. 1). In our study, the highest concentrations were recorded during the *Microcystis*-bloom expansion period (lasting from September to November) and decay stage (lasting from December to January) (Fig. 1 and Table S1). MCs were released along with the massive proliferation of bloom-forming *Microcystis aeruginosa* and during cell senescence and lysis. The highest concentration recorded during the bloom season was 44.12 μg L^−1^ (November), while that of the decay stage was 38.56 μg L^−1^ (December). In 2005, the ELISA analysis of MCs revealed a maximum of 60 and 94.40 μg L^−1^ (September and December, respectively) in irrigation water during the bloom formation and decay periods, respectively, in the Lalla Takerkoust region (El Ghazali et al. 2011). MCs persist in water for up to 251 days and could induce food chain contamination while consuming contaminated agrifood, such as crop plants and farm animals watered with MC-containing water (Zastepa et al. 2014; Redouane et al. 2021b). In accordance with our findings, MCs were generally less than 45 μg L^−1^, as reported elsewhere in previous investigations (Abdullahi et al. 2022; Mohamed et al. 2022). Previous research, however, found MC concentrations of up to 276 and 600 μg L^−1^ in lake waters used for crop irrigation (Xiang et al. 2019; Bakr et al. 2022).

### Health risk upon exposure to MC-containing water

To the best of our knowledge, there are a few papers in the recent relevant literature dealing with livestock exposure to MC-containing water and fodder, among others, the findings reported by Redouane et al. (2021a, b). Health risk evaluations of cyanotoxins in water have focused more on humans than farm animals during the last decade. Therefore, we attempted to assess the risk level for livestock and poultry upon the consumption of MC-polluted water sourced from the eutrophic reservoir of Lalla Takerkoust (the same water used for crop irrigation). The monitoring of MCs in water revealed concentrations higher than the safety limits recommended for farm animals (Table S2), showing moderate to high health risks upon consumption (Fig. 2). The risk level was very high in the water during the bloom and decay periods, during which MC concentrations were 11-fold higher than the safety limits for both cattle and sheep and were 14- and 19-fold higher for poultry and horses, respectively (during November). Moreover, horses and poultry were the most vulnerable farm animals, being exposed to a high-risk level (RQ > 1) when drinking MC-polluted water during all monitored months (Fig. 2). More recently, Redouane et al. (2021b) investigated the health risk related to the consumption of MC-containing fodder by cattle. They concluded that wheat-based fodder was of high health risk upon consumption, with up to a 7-fold increase compared with the recommended reference dose. Furthermore, it was reported that lethal MC levels in raw water were responsible for cattle toxicosis by causing severe hepatic damage. In addition, MCs were detected in the rumen content of one of the mortalities (Dreher et al. 2019). Overall, exposure to MCs could disrupt liver functions in cattle and thus affect the animal’s performance and growth, resulting in a major economic loss in the beef and dairy industries (Badar et al. 2017).

A public health concern has been raised about the potential bioaccumulation of MCs in animals used in agrifood and dairy production. To date, there are no official reports regarding MC accumulation in livestock and poultry, including the milk and eggs they produce. However, Orr et al. (2001, 2003) have investigated the impact of drinking cyanobacteria-blooming water (10^5^ cells of MC-producing *M. aeruginosa*) on dairy and beef cattle. Based on the data they obtained, the MCs detected in milk and liver tissues showed very low concentrations, posing no harm upon consumption. Still, further studies must be carried out to fill the knowledge gap regarding MC accumulation in meat and dairy products sourced from region-related overgrowths of toxic cyanobacteria.

Furthermore, MC-polluted irrigation water is also used as recreational water to fill swimming pools in the Lalla Takerkoust region, and thus, human health risks are likely to occur upon body contact and accidental ingestion of MCs. Monthly-average concentrations of MCs exceeded the WHO safety limit set at 1 μg L^−1^ in drinking water upon long-term exposure (up to a 44-fold increase). Moreover, toxin content during the bloom and decay periods outstripped WHO guideline limits set at 12 μg L^−1^ upon short-term exposure (up to a 4-fold increase) and 20 μg L^−1^ in recreational water (up to a 2-fold increase) (Table S4) (WHO 2020). Skin cells are the most exposed to MCs during recreational activities, which can result in the disruption of skin keratinocyte migration (chiefly the MC-LR variant), as proven by Kozdȩba et al. (2014). Furthermore, the inhalation of aerosolized MCs is a potential route of exposure during recreation (Backer et al. 2010), posing a serious health hazard since MCs may be responsible for lung carcinogenesis (Apopa et al. 2018).

### Microcystin accumulation in fruit crops

**Fig. 3:**
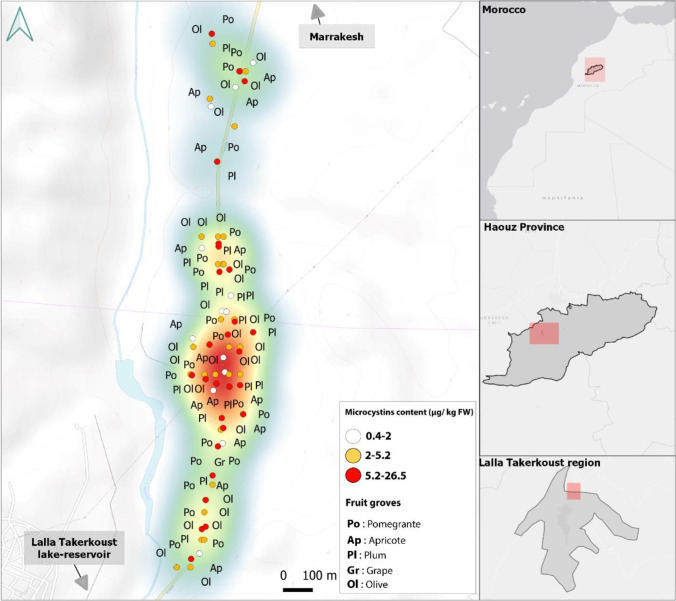
Distribution of microcystin concentrations (μg kg^−1^, fresh weight) in fruit crops irrigated with microcystin-containing water in the Lalla Takerkoust agricultural region. This distribution is represented by red, orange, and white dots on the map. The increasingly contaminated zones are ordered as follows: red > orange > yellow > green > blue

Data about MC accumulation in crop plants at environmentally realistic concentrations in irrigation water are scarce. Moreover, no previous studies have been conducted on MC accumulation in the edible parts of fruit trees. All investigated fruits (pomegranate, apricot, plum, grape, and olive) in the Lalla Takerkoust agricultural perimeter were found to accumulate MCs in the fresh and dried forms of edible parts. Highly contaminated crops (5.2-26.5 μg kg^−1^ FW) were shown by a red zone, as depicted in Fig. 3, in which the most MC-accumulating fruits (pomegranate and olive) were cultivated. The more the zone was contaminated, the more it was transitioning from blue to red colors (red > orange > yellow > green > blue). Apricot and plum crops were mainly concentrated in the green and blue zones (1.25-2.04 μg kg^−1^ FW). MC concentrations varied significantly among fruit crops (*p* < 0.05) ranging from 0.44 to 26.49 μg kg^−1^ in fresh fruits and from 0.10 to 7.20 μg kg^−1^ in dried fruits (only apricot, plum, and grape are consumed in dried form) (Table 1). This shift in MC content within different fruit crops was attributed to extrinsic factors such as soil features and intrinsic factors such as transpiration-driven uptake and toxin translocation processes (Corbel et al. 2016; Lee et al. 2017; Redouane et al. 2019), besides the variability of irrigation methods and quotas (Xiang et al. 2019). In our investigation, pomegranate was the most MC-accumulating fruit, reaching 26.49 μg kg^−1^ (fresh weight), followed by olive with a total content of 5.32 μg kg^−1^ (fresh weight) (Table 1). Pomegranate and olive crops were irrigated over bloom formation and decay periods during the fructification season. Hence, MCs were extensively released into the water following cyanobacterium-cell death and lysis.

**Table 1:** Concentrations of microcystins in fresh and dried edible parts of fruits

		Pomegranate ^b^	Apricot	Plum	Grape	Olive^b^
Total MCs (μg kg^−1^)	Fresh	26.49 ± 8.31	1.25 ± 0.24	2.04 ± 0.45	0.44 ± 0.03	5.32 ± 0.18
	Dry	***	7.20 ± 0.85	7.17 ± 0.39	0.10 ± 0.02	***
	D/F ^a^	***	5.79 ± 2.54	3.55 ± 0.86	0.24 ± 0.04	***

^a^D/F: microcystin ratio between fruit dry parts and their corresponding fresh parts

^b^Pomegranate and olive are only consumed in fresh form

Previous studies reported on MC accumulation in field vegetables (1.4–381.4 μg kg^−1^) that were primarily irrigated with MC-containing water at environmentally realistic concentrations (< 1 to 514 μg L^−1^) during the prolific growth of bloom-forming cyanobacteria (Cao et al. 2018; Xiang et al. 2019). Similarly, MC occurrence was investigated in market vegetables and found to accumulate at very high concentrations (0.17–4.49 μg g^−1^) after being irrigated with MC-polluted water (6.25 μg L^−1^) during the dry season (Chia et al. 2015; Chia et al. 2019). Overall, a dry season month is defined when its average precipitation is below 60 mm (Peel et al. 2007). The Lalla Takerkoust region in our study is a semi-arid area with a long dry season and a monthly-average precipitation of less than 50 mm over the last 4 years (NASA 2021) (Fig. S2). The dry climate in arid and semi-arid regions is usually linked with rainfall scarcity and the expansion of toxic cyanoblooms, which increases the outflow of MC-containing water for irrigation (Barros et al. 2019; Zuccarello et al. 2021). In this regard, a previous study was conducted on the occurrence of MCs in vegetable crops grown under realistic exposure scenarios in arid and semi-arid climates (Saudi Arabia). These crops were found to accumulate MCs in the range of 0.07–1.6 μg g^−1^ (fresh weight) when irrigated with MC-contaminated water sourced from contaminated wells (0.6–1.6 μg L^−1^) and rainwater ponds (0.65–2.3 μg L^−1^) nearby (Mohamed and Al Shehri 2009).

### Human health risk upon exposure to MC-accumulating fruits

**Fig. 4:**
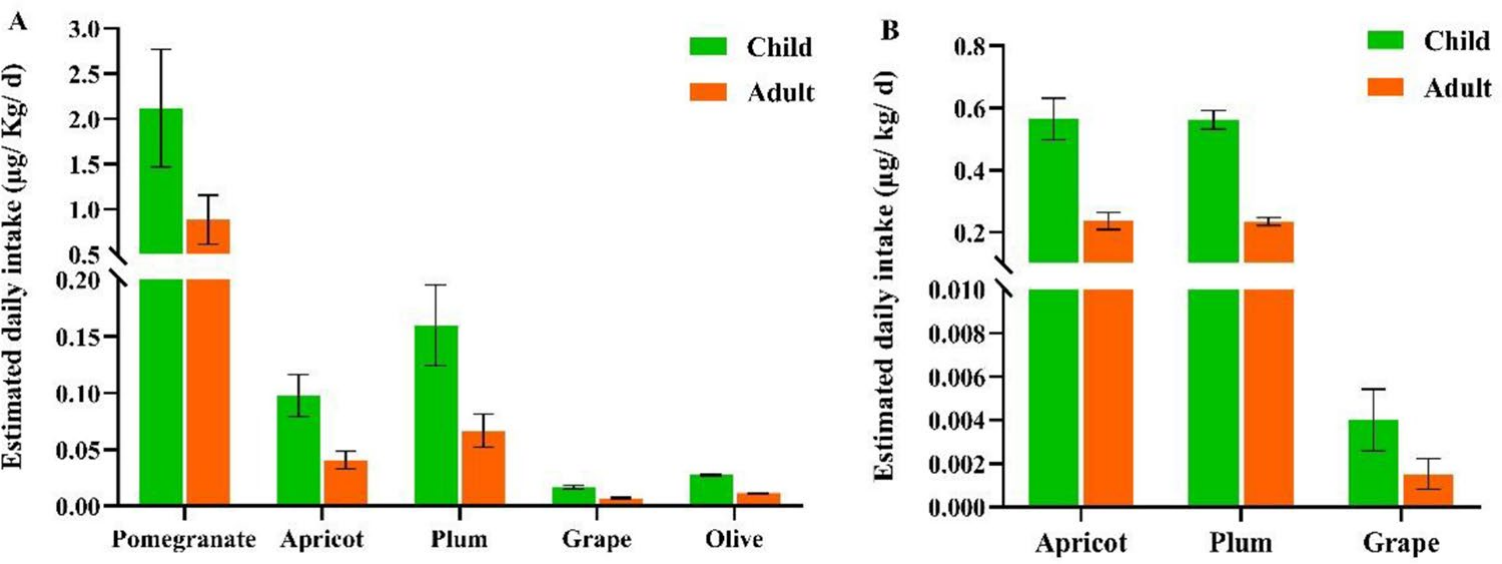
Estimated daily intake of microcystins in fresh (**A**) and dried fruits (**B**) (μg eq MCs kg bw^−1^ d^−1^) for adults and children. The World Health Organization has set a limit value of 0.04 μg kg^−1^ d^−1^ (WHO 2020). Only apricot, plum, and grape were largely consumed in dry form

**Fig. 5:**
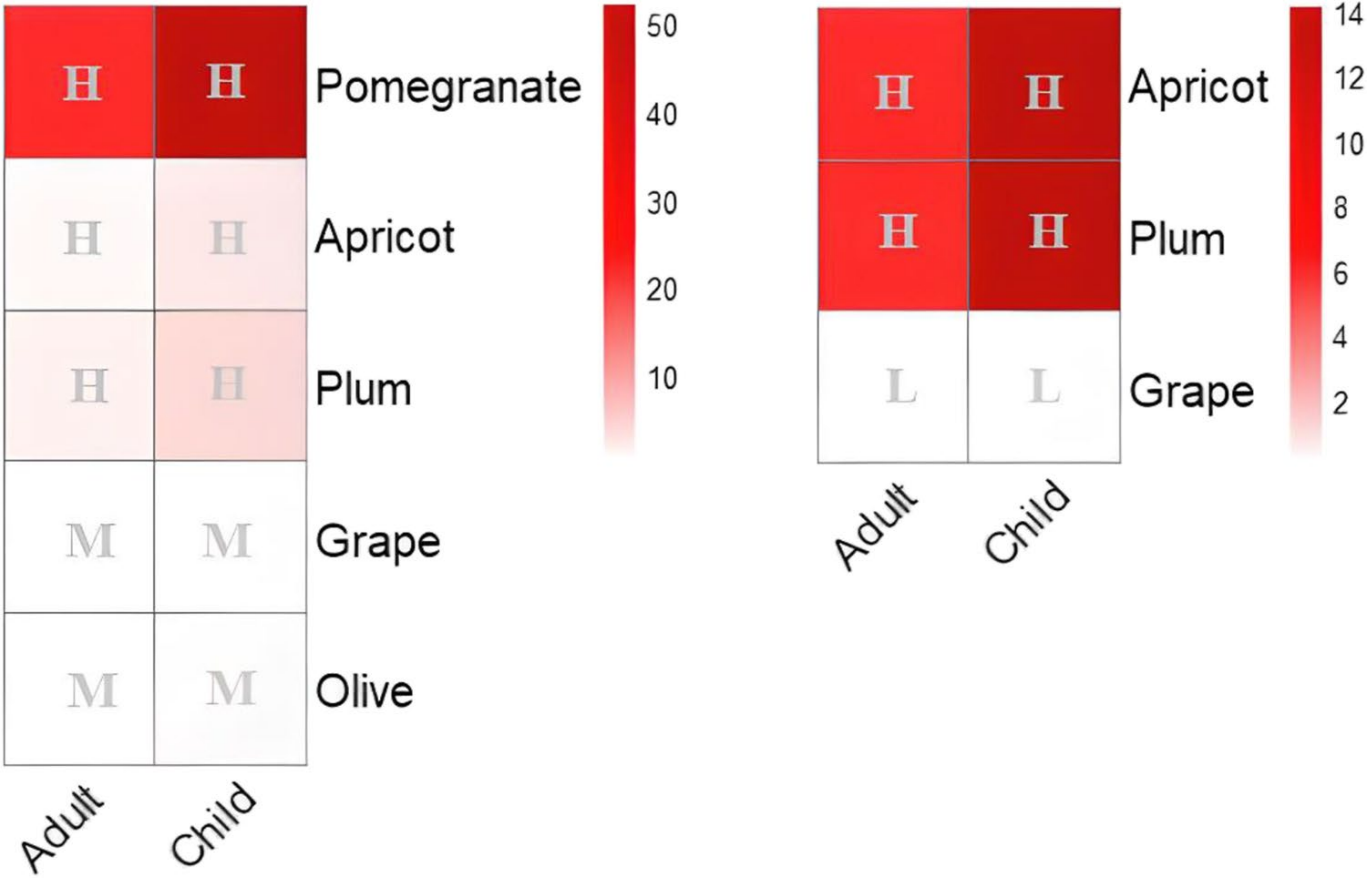
Heatmap of risk quotients and risk levels (H: high, M: moderate, L: low) related to microcystin consumption in fruit crops for both adults and children. Left: fresh fruits; right: dry fruits

There were a few reports of health risk assessment regarding MC accumulation in plant crops and related irrigation water at environmentally relevant concentrations, including the studies conducted by Cao et al. (2018), Chia et al. (2019), Xiang et al. (2019), and Levizou et al. (2020). Residents in the Lalla Takerkoust town and Marrakesh region could be exposed to MCs when consuming MC-accumulating crops. Therefore, we calculated the EDI of MCs in mature fruits to assess the health risk related to their consumption. For adults, the EDI of MCs in fresh fruits ranged from 0.01 to 0.88 μg kg bw^−1^ d^−1^, and for children, it ranged from 0.02 to 2.12 μg kg bw^−1^ d^−1^ (Fig. 4A). As for dried fruits, the EDI ranged from 0.002 to 0.24 μg kg bw^−1^ d^−1^ for adults and from 0.004 to 0.56 μg kg bw^−1^ d^−1^ for children (Fig. 4B). The EDI of MCs for both adults and children exceeded the TDI (0.04 μg kg bw^−1^ d^−1^) in pomegranate by 22- and 53-fold increases, respectively, posing a very high-risk level upon its consumption (RQ > 1). It was also 2- and 4-fold higher in fresh plums for both adults and children, respectively, indicating a high health risk as well. Furthermore, fresh apricots exceeded the TDI by a 2-fold increase for children only. As for olives and grapes (fresh), they showed a moderate-risk level (0.1 ≤ RQ ≤ 1) for both adults and children. Moreover, dried plums and apricots exhibited a similar risk level, 2- and 6-fold higher than the TDI for both adults and children, respectively (Fig. 5). When apricot and plum edible tissues were dried, MCs were concentrated 6 and 4 times, respectively, posing a high health concern upon consumption (Table 1). However, dried grapes surprisingly showed a low health risk (RQ < 0.1) for both adults and children (Fig. 5). It should be pointed out that health risk levels could fluctuate across years due to the varying daily intakes of fruits among adults and children. Moreover, the ever-increasing scarcity of rainfall implies a growing demand for irrigation water in MC-occurring lakes and reservoirs and thus could increase MC accumulation in edible crops.

Xiang et al. (2019) found EDI values ranging from 0 to 0.73 μg kg bw^−1^ d^−1^ in vegetable crops irrigated with cyanobloom-occurring waters (0.3 to 514.4 μg L^−1^ of MCs), with children a bit higher than adults. They reported a high health risk upon consumption of leafy vegetables (chiefly celery) with EDI values above the recommended TDI, whereas root vegetables and some fruit vegetables showed moderate and low health risks. In another study carried out by Chia et al. (2019), EDI values of MCs were higher than the WHO safety limit upon consumption of MC-accumulating vegetables sold in markets during the dry season. The highest EDI of a 60-kg adult was 3.19 μg kg bw^−1^ d^−1^ for green amaranth, 2.94 μg kg bw^1^ d^−1^ for lettuce, and 1.41 μg kg bw^−1^ d^−1^ for cabbage. The highest values for a 25-kg child were 1.91 μg kg bw^−1^ d^−1^ for green amaranth, and 1.77 μg kg bw^−1^ d^−1^ for lettuce. More recently, Bakr et al. (2022) detected naturally occurring MCs (produced by *M. aeruginosa* and *Oscillatoria limnetica*) in water (45–600 μg L^−1^) used to irrigate leafy vegetables in farmlands close by. EDI values were much higher than the TDI, recording up to 3.63 kg bw^−1^ d^−1^ and 4.36 for adults and children, respectively. Once ingested, MCs could bioaccumulate inside the body and thus cause adverse health effects on various vital organs, including the liver, small intestine, brain, kidney, lung, and heart (Massey et al. 2018). Over recent years, several risk assessment studies have been carried out on edible vegetables under lab and greenhouse conditions being irrigated with unnaturally occurring concentrations of MCs (Bittencourt-Oliveira et al. 2016; Cordeiro-Araújo et al. 2016; Zhu et al. 2018; Xiang et al. 2020). Therefore, these studies, including ours, emphasize the health risk linked to environmentally relevant concentrations of MCs in irrigation water and crop plants, mainly during the expansion of MC-producing cyanobacteria. Furthermore, the risk assessment in our study was based on the TDI of MC-LR set by WHO since it was dominant in the *Microcystis* bloom occurring in the Lalla Takerkoust reservoir. Moreover, the toxicity of the MC-LR variant is more pronounced, while that of others such as MC-RR is still unclear (Gupta et al. 2003; Díez-Quijada et al. 2019). Therefore, future studies must address the issue of the difference in toxicity between MC congeners and set a TDI similar to that of MC-LR to fully and accurately estimate the health risk. Overall, there is an urgent need to develop new monitoring strategies based on environmentally realistic concentrations of MCs in edible crops and set up bioremoval tools to rule out their adverse health effects.

## Conclusions

Our findings throughout this study provide insights into MC accumulation in edible fruits and related health risks under an environmentally realistic exposure scenario. They demonstrated the transfer of MCs from irrigation water to edible fruits in the areas affected by toxic cyanoblooms. Pomegranate and olive were the most MC-accumulating fruits due to toxin uptake from MC-polluted water during the blooming and decay periods. Furthermore, MCs pose a health risk upon the consumption of contaminated pomegranates, plums, and apricots. These results point to the urgent need to monitor MC levels in irrigation water and fruit crops from grove to market. Likewise, MCs pose a great health risk to livestock and poultry being watered with MC-polluted water, chiefly during the blooming and decay periods. MC toxicosis could lead to health problems and eventual deaths in farm animals, and thus cause an economic loss in dairy and beef production. Furthermore, MCs may accumulate in poultry- and livestock-based foods commercialized for human consumption, and therefore, we strongly suggest implementing new policies strategies for monitoring their levels in these products. Overall, further remediation strategies should pay more attention to MC removal from raw water used in farming practices to stave off their uptake by plant crops and farm animals, chiefly in water-scarce and bloom-affected areas. Furthermore, we need to better understand the MC toxicity of different variants, primarily the most prevalent ones, for more accurate and certain results in health risk assessment.

## Supplementary information

ESM 1 (PDF 492 kb)

## Data Availability

Data regarding the average daily intake of vegetables for children and adults related to Morocco (used to calculate the EDI of MCs) can be found on the Food and Agriculture Organization of the United Nations’ website at http://www.fao.org/faostat/en/?#data/FBS, accessed on June 13, 2022.
