# Supplementary material for: Health risk assessment of lake water contaminated with microcystins for fruit crop irrigation and farm animal drinking
Source: Environ Sci Pollut Res Int. 2023 Jun 9;30(33):80234–44. doi: 10.1007/s11356-023-27914-1 (PMC10344998; doi:10.1007/s11356-023-27914-1)
Supplement: Supplementary file 1 — (PDF 492 kb) [file 11356_2023_27914_MOESM1_ESM.pdf]

Environmental Science and Pollution Research

Supplementary data for:

Health risk assessment of lake water contaminated with microcystins for fruit crop irrigation and farm animals drinking

El Mahdi Redouane[1,2], Zakaria Tazart[1], Majida Lahrouni[1], Richard Mugani[1], Sara Elgadi[3,4], Hamza Zine[5], Soukaina El Amrani Zerrifi[1,6], Mohammed Haida[1], José Carlos Martins[2], Alexandre Campos[2], Khalid Oufdou[3], Vitor Vasconcelos[2,7] and Brahim Oudra[1]

[1] Water, Biodiversity and Climate Change Laboratory. Faculty of Sciences Semlalia, Cadi Ayyad University, 40000 Marrakech, Morocco.

[2] CIIMAR, Interdisciplinary Centre of Marine and Environmental Research, Terminal de Cruzeiros do Porto de Leixões, Av. General Norton de Matos, s/n, 4450-208 Matosinhos, Portugal.

[3] Laboratory of Microbial Biotechnologies, Agrosciences, and Environment (BioMAgE), Labeled Research Unit-CNRST N°4, Faculty of Sciences Semlalia, Cadi Ayyad University, 40000 Marrakech, Morocco.

[4] Laboratory of Agro. Food Technology and Quality, Regional Center for Agronomic Research of Marrakech, National Institute of Agronomic Research (INRA), 40000 Marrakech, Morocco.

[5] Geology and Sustainable Mining Institute (GSMI), Mohammad VI Polytechnic University, 43150 Ben Guerir, Morocco.

[6] Higher Institute of Nurses Professions and Health Techniques of Guelmim, 81000 Guelmim, Morocco

[7] Department of Biology, Faculty of Sciences, University of Porto, 4169-007 Porto, Portugal.

Corresponding author: Vitor Vasconcelos, vmvascon@fc.up.pt.

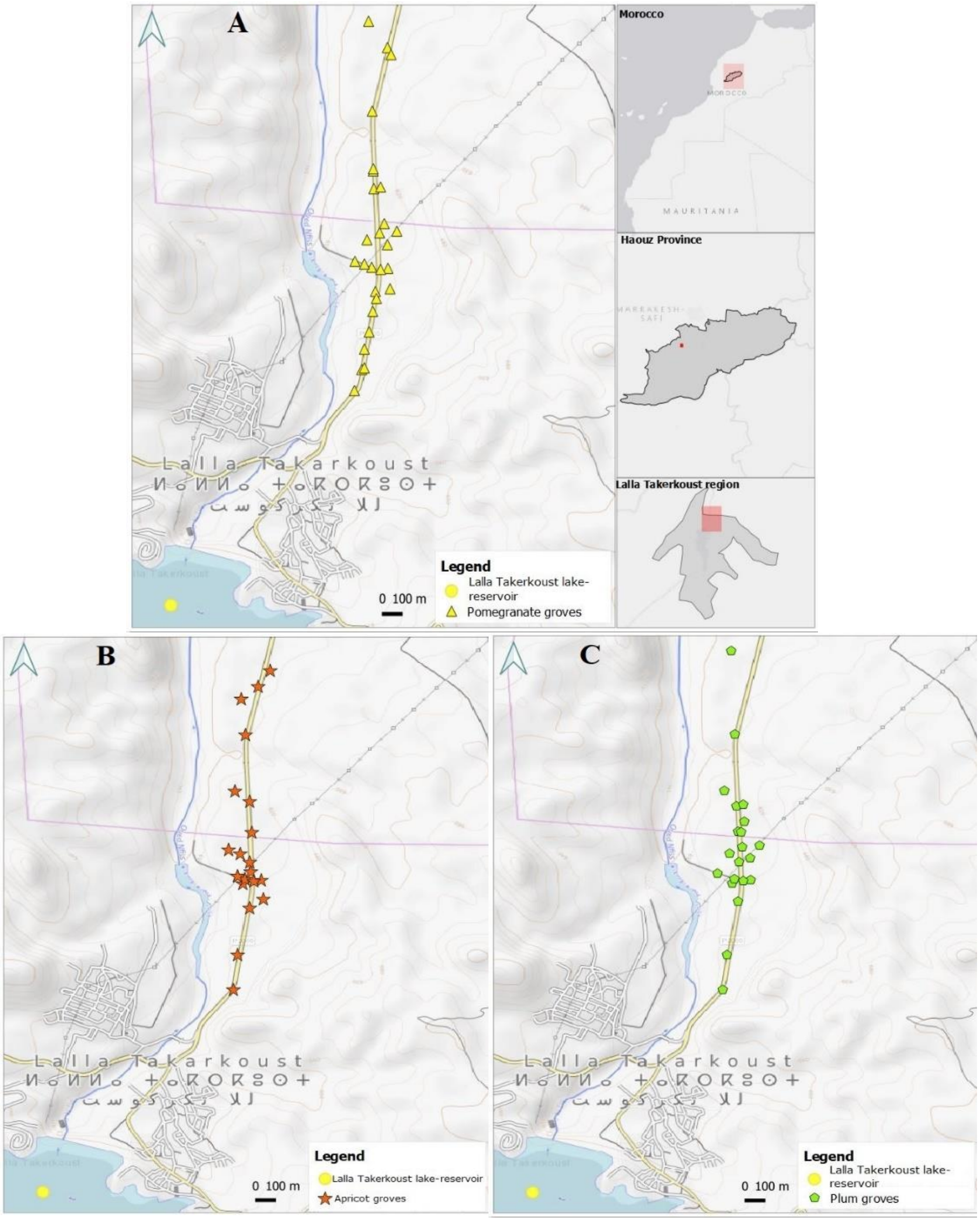
A
Morocco
MOROCCO
MAURITANIA
Haouz Province
MARRAKESH-SAFI
Lalla Takerkoust region
Lalla Takarkoust
Legend
Lalla Takerkoust lake-reservoir
Pomegranate groves
0 100 m
B
Lalla Takarkoust
Legend
Lalla Takerkoust lake-reservoir
Apricot groves
0 100 m
C
Lalla Takarkoust
Legend
Lalla Takerkoust lake-reservoir
Plum groves
0 100 m

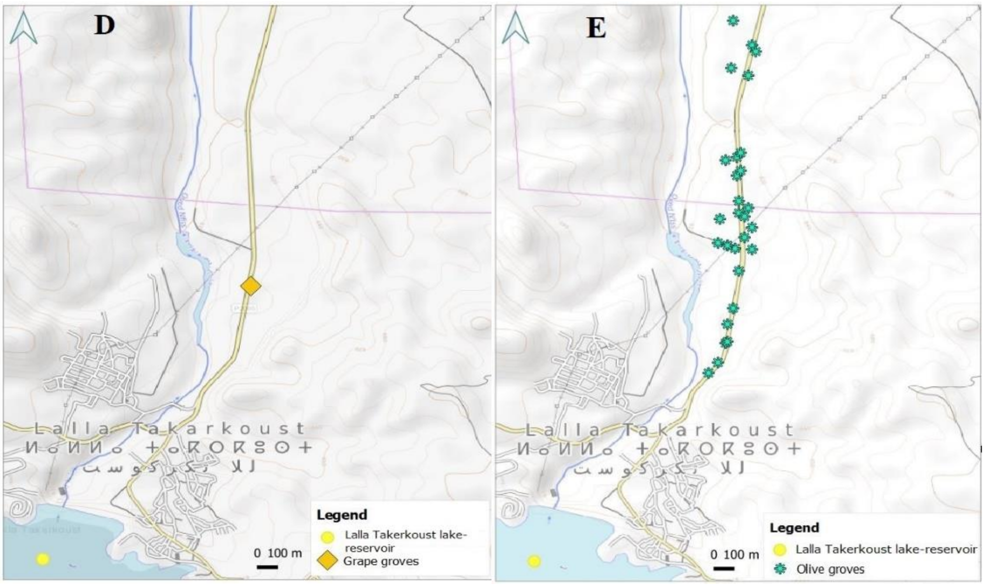

Fig. S1 Sampling sites of fruit crops in the Lalla Takerkoust agricultural perimeter being irrigated from the Lalla Takerkoust lake-reservoir affected by *Microcystis*-bloom, Marrakesh, Al Haouz province, Morocco. (A) pomegranate, (B) apricot, (C) plum, (D) grape, (E) olive

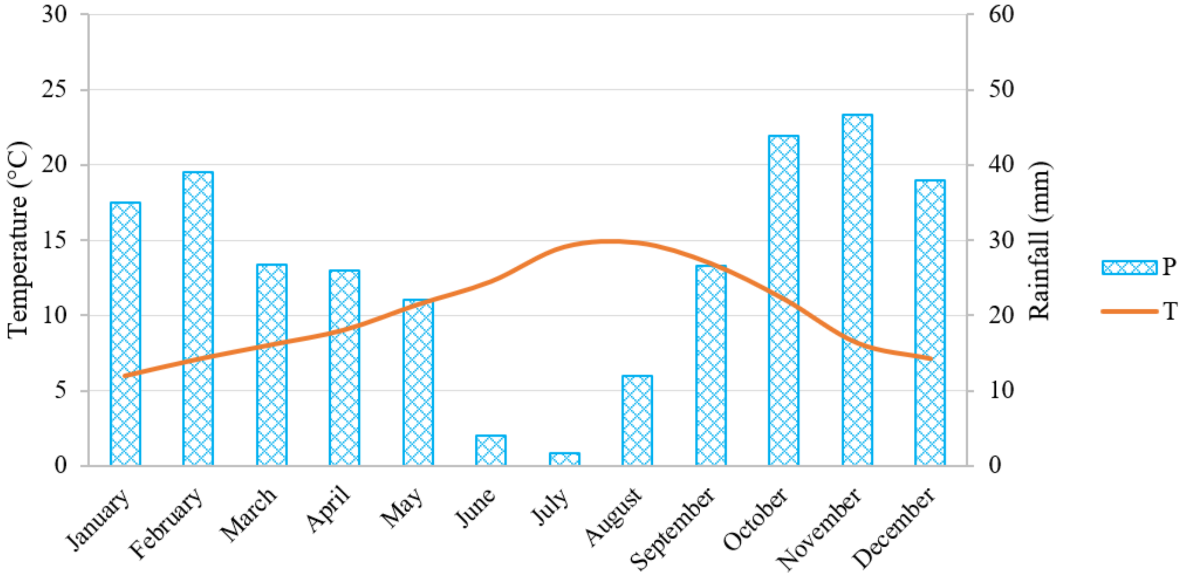

Fig. S2 Ombrothermic diagram of the Lalla Takerkoust region (means of years 2018-2021). The temperature and precipitation data used for the construction of the ombrothermic diagram were obtained from NASA's official site (NASA, 2021)

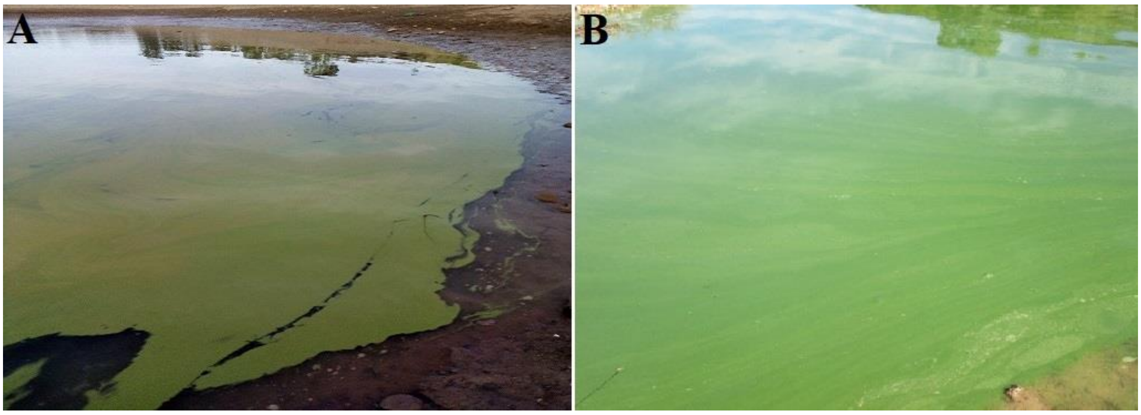

Fig. S3 *Microcystis* bloom in the Lalla Takerkoust lake-reservoir, Marrakesh, Morocco; (a) September 2019, (b) September 2017 (These photos have been taken by this paper's co-authors)

Table S1 Bloom formation and decay periods (high release of MCs into the water). The asterisks (*****) indicate the harvest months of the studied fruit crops in the Lalla Takerkoust region. Months of bloom formation/decay period and harvest stage were determined based on our observations during the last 4 years (2019-2022)

	January	February	March	April	May	June	July	August	September	October	November	December
	Bloom decay								Bloom formation			Bloom decay
Apricot					*	*						
Plum						*	*					
Grape							*	*				
Pomegranate								*	*	*		
Olive											*	*

Table S2 Average body weights of poultry and livestock and their respective daily intake of water, in addition to tolerable daily intakes (TDI) of microcystins according to the Australian and New Zealand Environmental and Conservation Council (ANZECC) (ANZECC, 2000)

Animal	Body weight (kg)	water intake (L d^{-1})	TDI (µg L^{-1})
Cattle	800	85	4.2
Sheep	100	11.5	3.9
Chickens	2.8	0.4	3.1
Horses	600	70	2.3

Table S3 Average amount of consumed fruits in Morocco over 2019 during the harvest season according the Food and Agriculture Organization of the United Nations (FAO, 2019)

Area	Ref	Year	Item	Kg/capita/d
Morocco	FAO	2019	Olives (including preserved)	0.13
			Grapes and products	0.94
			Other fruits	1.96

Table S4 Monthly content of microcystins during 2019 in irrigation water (μg L^{-1}) and related risk quotients for recreation (swimming) and long-/short-term exposures via accidental ingestion (during swimming).

			January	February	March	April	May	June	July	August	September	October	November	December
MCs (μg L^{-1})			19.09 ± 1.51	11.40 ± 0.63	3.94 ± 0.52	5.21 ± 0.20	9.96 ± 0.48	3.33 ± 0.26	7.76 ± 2.48	7.64 ± 1.80	15.53 ± 2.85	37.47 ± 1.09	44.12 ± 1.67	38.56 ± 13.08
RQ	Accidental ingestion	LT	**19.09 ± 1.51**	**11.40 ± 0.63**	**3.94 ± 0.52**	**5.21 ± 0.20**	**9.96 ± 0.48**	**3.33 ± 0.26**	**7.76 ± 2.48**	**7.64 ± 1.80**	**15.53 ± 2.85**	**37.47 ± 1.09**	**44.12 ± 1.67**	**38.56 ± 13.08**
		ST	**1.59 ± 0.13**	0.95 ± 0.05	0.33 ± 0.04	0.43 ± 0.02	0.83 ± 0.04	0.28 ± 0.02	0.65 ± 0.21	0.64 ± 0.15	**1.29 ± 0.24**	**3.12 ± 0.09**	**3.68 ± 0.14**	**3.21 ± 1.09**
	Recreation		0.95 ± 0.08	0.57 ± 0.03	0.20 ± 0.03	0.26 ± 0.01	0.50 ± 0.02	0.17 ± 0.01	0.39 ± 0.12	0.38 ± 0.09	0.78 ± 0.14	**1.87 ± 0.05**	**2.21 ± 0.08**	**1.93 ± 0.65**

RQ: risk quotient representing the factor exceeding the WHO safety limit.
LT: long-term exposure to MCs; WHO safety limit is set at 1 μg L^{-1} (WHO, 2020).
ST: short-term exposure to MCs; WHO safety limit is set at 12 μg L^{-1} (WHO, 2020).
WHO safety limit for recreational water is set at 20 μg L^{-1} (WHO, 2020).
Bolded values correspond of high-risk level (RQ > 1) based on RQ values (RQ = MCs in water (μg L^{-1})/ corresponding WHO safety limit (μg L^{-1})).

References

ANZECC (2000) Water Quality Guidelines in Livestock Drinking Water Guidelines. Australian and New Zealand Environment and Conservation Council. https://www.waterquality.gov.au/anz-guidelines/resources/previous-guidelines/anzecc-armcanz-2000. Accessed 10 January 2021.

FAO (2019) New Food Balances. Food and Agriculture Organization of the United Nations. http://www.fao.org/faostat/en/?#data/FBS. Accessed 13 June 2022.

NASA (2021) NASA POWER global meteorology at NASA POWER Prediction Of Worldwide Energy Resource. https://power.larc.nasa.gov/#contact. Accessed 02 July 2022

WHO (2020) Cyanobacterial toxins: microcystins. Background document for development of WHO Guidelines for drinking-water quality and Guidelines for safe recreational water environments. World Heal Organ. https://apps.who.int/iris/bitstream/handle/10665/338066/WHO-HEP-ECH-WSH-2020.6-eng.pdf. Accessed 11 May 2021
